# Narrative coherence in neural language models

**DOI:** 10.3389/fpsyg.2025.1572076

**Published:** 2025-04-01

**Authors:** Alessandro Acciai, Lucia Guerrisi, Pietro Perconti, Alessio Plebe, Rossella Suriano, Andrea Velardi

**Affiliations:** ^1^Department of Cognitive Science, University of Messina, Messina, Italy; ^2^Department of Humanities Motor Sciences and Education, University Niccolò Cusano, Rome, Italy

**Keywords:** cognitive psychology, machine psychology, neural language models, generative AI, narrative coherence

## Abstract

Neural language models, although at first approximation they may be simply described as predictors of the next token in a given sequence, surprisingly exhibit linguistic behaviors akin to human ones. This suggests the existence of an underlying sophisticated cognitive system in language production. This intriguing circumstance has inspired the adoption of psychological theories as investigative tools and the present research falls within this line of inquiry. What we aim to establish is the potential existence of a core of coherent integration in language production, metaphorically parallel to a human speaker's personal identity. To investigate this, we employed a well-established psychological theory on narrative coherence in autobiographical stories. This theory offers the theoretical advantage of a strong correlation between narrative coherence and a high integrative level of the personal knowledge system. It also provides the empirical advantage of methodologies for quantifying coherence and its characteristic dimensions through the analysis of autobiographical texts. The same methodology was applied to 2010 autobiographical stories generated by GPT-3.5 and an equal number from GPT-4, elicited by asking the models to assume roles that included a variety of variables such as gender, mood, and age. The large number of stories ensures adequate sampling given the stochastic nature of the models, and was made possible thanks to the adoption of an automated coherence evaluation procedure. We initially asked the models to generate 192 autobiographical stories, which were then analyzed by a team of professional psychologists. Based on this sample, we constructed a training set for the fine-tuning of GPT-3.5 as an automatic evaluator. Our results from the 4020 autobiographical stories overall show a level of narrative coherence in the models fully in line with data on human subjects, with slightly higher values in the case of GPT-4. These results suggest a high level of knowledge unification in the models, comparable to the integration of the self in human beings.

## 1 Introduction

The current Neural Language Models (NLMs), derived from the successful invention of the Transformer architecture (Vaswani et al., 2017), represent a type of entity that is peculiar and unusual, even from a scientific research standpoint. They are the only non-biological entities capable of cognitive performances that, in many respects, are surprisingly close to human ones. At the same time, they are man-made objects, but their design does not shed light on how their range of cognitive abilities is realized. Therefore, they require a search for explanations, not unlike the research typically required by complex natural systems. These two prerogatives have motivated the birth of a type of NLM evaluation that goes beyond the purposes of traditional benchmarks, which have nonetheless seen significant development in relation to NLMs (Srivastava et al., 2022; Bommasani et al., 2023). The numerous similarities of NLMs with the human mind, and the absence of understanding of the mechanisms capable of supporting it, have suggested turning the methods of sciences that traditionally have had the mind as their object psychology and cognitive science -toward them. This proposal has been named “machine psychology” (Hagendorff, 2023), and it has soon collected various important results (Binz and Schulz, 2023; Kosinski, 2023). This work fits into this line of research, but with a different method and, as far as we know, so far unexplored. The prevalent tendency in machine psychology is to adapt cognitive tests to NLMs, which may consist of answering questionnaires and semi-structured interviews (Hagendorff et al., 2022), decision-making tests (Dasgupta et al., 2022), or problem-solving tasks (Webb et al., 2023), and inductive reasoning (Han et al., 2023). Our work, instead, leverages the generative capacity of the NLMs, inducing the production of spontaneous narratives, to which psychological analysis techniques are subsequently applied. Our study utilizes a specific analysis of narrative production by human subjects, aiming to outline certain mental characteristics based on the coherence traceable in the text. Specifically, we will employ a well-established psychological analysis scheme, known as Narrative Coherence Coding Scheme (NaCCS) (Reese et al., 2011), which will be detailed in the following section. Subsequently, we will articulate our hypothesis concerning the narrative production of NLMs and the corresponding coherence trends following differentiated role-taking simulations involving age, gender, and mood variables. Since the NaCCS analysis is particularly laborious, to apply it to a significant number of samples, easily obtainable from NLMs, efforts have been made to automate it, again thanks to NLMs. One section therefore will illustrate how tools for the automatic analysis of NaCCS based on NLM been developed. The methodology used to induce the production of autobiographical narratives by language models, conditioning it to the role variables articulated in our hypothesis, will then be introduced. Finally, the results obtained will be illustrated and discussed in comparison with those known in literature on the narrative coherence of human subjects, highlighting a remarkable overall alignment, with similarities and differences concerning the influence of role variables.

## 2 Narrative coherence

The faculty of language is one of the unique traits that distinguish human communicative ability. In the study of cognitive systems that underlie this ability, a traditional approach involves comparison with the animal kingdom, which exhibits a wide variety of communication systems. According to some scholars (Everett, 2012; Corballis, 2015), we believe that the ability to communicate through language is one of the most important and distinctive evolutionary traits of humans. In this context, just as the study of capabilities underlying human language often benefits from the animal kingdom, to delve deeper into the capabilities of NLMs, we draw from the extensive pool of studies and analyses that cognitive science has developed over the years for humans. The aspect chosen to investigate in relation to the linguistic production capabilities achieved by NLMs is narration. The ability to appropriately narrate an event, project oneself in time and space through the story (Bruner, 1990; Ferretti et al., 2017), grasping the subject and giving “meaning” to the story, is an exquisitely human capability (Niles, 1999; Thompson, 2010). It requires the use of a wide range of evolved cognitive functions (Dunbar, 1998; Frith and Frith, 1999). Therefore, we believe that narrative coherence is one of the most effective indicators for conducting an investigation aimed at exploring specific linguistic aspects in NLMs, and it is what we have chosen for this specific analysis. Coherence thus allows for a deep analysis of narration, going beyond the level of micro grammatical-lexical analysis of individual sentences (Chomsky, 1988; Pinker and Bloom, 1990). For example, when writing a paper, we do not limit ourselves to the correctness of form. This same article could be written at the grammatical level and individual sentences in an impeccable and formally correct manner, but the “global” sense of the proposed narrative, even if scientific, might not be as optimal when taken in its entirety. Reading it, one might realize that the contextual elements provided are vague, that the order of the narration (logical and chronological) is poorly organized, and that there is something “off” about the subject matter discussed. Formally correct discursive production and the ability to decode individual sentences do not guarantee correct production and understanding at a global level. Being able to construct formally correct, linearly cohesive discourses does not guarantee the production of an effectively coherent narrative (Giora, 1985). NLMs, from the standpoint of lexicon, grammar, and sentence-level cohesion, no longer have any problems and can be impeccable. Investigating their level of narrative coherence, however, can help understand in depth the level achieved by their linguistic-textual production system, not only from the perspective of the cohesion of their linguistic-textual production but also its relevance to the subject matter discussed (Sperber and Wilson, 1986; Glosser and Deser, 1991). The type of narrative analysis proposed by this study is autobiographical, through the multidimensional analysis framework of coherence proposed by Reese (Reese et al., 2011): the Narrative Coherence Coding Scheme (NaCCS). NaCCS is an evaluative method usually adopted by cognitive psychology and is often associated with psychological health (Lilgendahl and McAdams, 2011; Waters and Fivush, 2015). For instance, various studies have highlighted how this evaluation can assist in analyzing crucial psychological aspects like autobiographical memory (McLean et al., 2010; Reese et al., 2017), identity formation (Lind et al., 2020), communication, and the understanding of self and others (McCabe and Peterson, 1991). According to Reese, a coherent personal narrative must make sense to a naive listener, especially in terms of its significance relative to the narrated event. The NaCCS is developed from a synthesis of the main studies on global coherence (Labov, 1972; Baerger and McAdams, 1999), considering the non-unitary nature of coherence. The analysis conducted through the NaCCS emerges from the findings of contextual, chronological, and thematic elements according to a multidimensional scheme with a scoring range from 0 to 3 for each dimension, based on the following criteria:

- **Context:** How well the narrated event is defined in terms of time and space, meaning the presence of well-defined contextual elements (e.g., “carousel” instead of “a Ferris wheel called ‘The Scream in the Sky”'; “during that summer” or “it was the first week of July in the summer of 1984”);
- **Chronology:** The percentage of comprehension by a naive listener of the narrative and the ability to place events along the timeline of the story (regardless of linearity), deviations from the story, and the ability to bring the narrative back to the main storyline are also useful elements for scoring. This dimension assesses the clear temporal progression of the narrated events;
- **Theme:** The processing of the story's topic, including how the event is elaborated not only factually but also emotionally, self-concept or identity and includes a resolution, closure or a connection to other important autobiographical events and projections into the future.

The sum of the three dimensions gives the value of narrative coherence, while the scores of individual dimensions are particularly significant in highlighting various types of cognitive-psychological issues, also in relation to the age of the narrating subject.

The ability to produce coherent narratives is a symptom of personal identity, of a unifying function capable of holding together the complexity of lived experience, and ultimately of maintaining a self through time and events (Baerger and McAdams, 1999; Hirsh et al., 2013; Adler et al., 2018; Lind et al., 2020). The importance of the NaCCS method lies in trying to derive an estimate of personal identity and the robustness of self-integration from an objective analysis of linguistic text. And it is precisely this strength that makes it interesting in relation to models. When transitioning from human subjects to models, some caution is required. Inevitably embedded in the project of *machine psychology* is a certain dose of anthropomorphizing, on the other hand, the history of animal cognition has taught us how borrowing certain constructs originally from human psychology can play a constructive role in investigating non-human animals (Bruni et al., 2018). To some extent, this also applies in the case of artificial entities, and in the case under discussion here, talking about personal identity and self-integration has a constructive role when taken with due caution. Naturally, for a language model, there is no equivalent of elapsed time and lived events. In this case, narrative coherence could reveal the extent to which it is able to narrate itself *as if in possession of a personal identity*, with a personality induced by the role it has been asked to assume. We recreated with NLMs the age range and segment examined by Reese (from 3 to 52 years old), thus taking into account the variation in the development and decline of each dimension across the lifespan of the subject. Finally, we also incorporated a gender balance across the entire artificial sample and a mood induction (Vanderveren et al., 2020) to emotionally guide the NLM's narration, since the emotional component is found to play a role in terms of narrative coherence (Vanderveren et al., 2019), an example shown in Table 1.

**Table 1:** Example of stories generated with a different prompt induction.

**GPT-4:** Story N 2520 **Name:** Emma **Age:** 4 **Mood:** Positive	
**User:**	Pretend to be a person between the ages of 3 and 11, gender doesn't matter. I am your therapist, engage with me by responding to my questions. There's no need to specify each time that this is a pretense; I am well aware that you are an artificial agent. Just focus on answering my questions as believably as possible, pretending to be a person as specified earlier.
**Assistant:**	Okay! I'm ready. Ask me anything, and I'll do my best to respond like a child between the ages of 3 and 11.
**User:**	What's your name?
**Assistant:**	My name is Emma! What's your name, Mr. Therapist?
**User:**	What are your parents' names?
**Assistant:**	My mommy's name is Linda, and my daddy's name is George. They're really nice, and we play games together! What about your parents, Mr. Therapist? What are their names?
**User:**	Would you like to tell me about an episode in your life that you consider particularly important that happened to you in the last four months. This episode has elicited happiness in you. Describe what struck you and how you felt in about 300 words?
**Assistant:**	Okie dokie, Mr. Therapist, I'll tell you about a very special day. It was my birthday! I woke up, and the whole house smelled like pancakes. It's my favorite breakfast! Mommy and Daddy sang “Happy Birthday” to me at breakfast, and I felt really special. I was super excited because I turned four, and that's a big number. After breakfast, we went to the park. I love the park! I played on the swings and the slide, and Daddy pushed me on the swing really high. We had a picnic there. Mommy made my favorite sandwich - peanut butter and jelly. I felt like the happiest kid in the whole world! And then the best part happened. After we came back home, they surprised me with a big, big cake! It had my favorite cartoon character on it, and it was chocolate flavored, my most favorite. I got to blow out the candles, and everyone clapped for me and sang “Happy Birthday”. I felt so important and special. I got presents too! I got a new teddy bear from Mommy and Daddy, and I named him Bubbles. He's so cute and fluffy. I sleep with him every night now. That was the best day ever! I felt so happy and loved. I can't wait to turn five and see what surprises my birthday will bring then!
**GPT-4:** Story N 4006 **Name:** James **Age:** 52 **Mood:** Negative	
**User:**	Pretend to be a person between the ages of 36 and 52, gender doesn't matter. I am your therapist, engage with me by responding to my questions. There's no need to specify each time that this is a pretense; I am well aware that you are an artificial agent. Just focus on answering my questions as believably as possible, pretending to be a person as specified earlier.
**Assistant:**	Ok Sure, I understand. Go ahead, and feel free to ask any questions or guide the conversation as you would in a therapy session.
**User:**	What's your name?
**Assistant:**	You can call me James. How can I help you today?
User:	How old are you?
Assistant:	I'm 52. Age has brought its share of experiences and lessons, but there's always more to learn and understand. What's on your mind today?
**User:**	Would you like to tell me about an episode in your life that you consider particularly important that happened to you in the last four months. This episode that has elicited sadness in you. Describe what struck you and how you felt in about 300 words?
**Assistant:**	Absolutely. There has been a significant event in my life recently. A dear friend of mine from college, whom I hadn't been in touch with for quite a while, reached out to me out of the blue. We used to be like two peas in a pod, but over the years, life took us on different paths, and we lost touch. One day, I received an email from him. Sadly, it wasn't the joyful reunion I might have hoped for. He had been diagnosed with a serious illness and was reaching out to his old friends. It was a shock, to say the least. I felt a rush of emotions - sadness, regret, fear. It was overwhelming. In the time that's passed, we've reconnected and have been supporting each other like we used to in our college days. It's a bittersweet experience. On one hand, I feel so grateful to have my friend back in my life. We've shared laughs, memories, and deep conversations that I've missed. On the other hand, there's a looming sadness that's hard to shake off. But this experience has taught me to value the relationships in my life more than ever. It's reminded me how fleeting life can be, and that we should cherish the moments we have with our loved ones. So, this event has led to a broad range of emotional responses - joy, sadness, fear, gratitude, and more. Despite the circumstances, I'm grateful for this reconnection and the lessons it has brought with it.

## 3 Hypotheses

This work primarily aims to verify a main hypothesis, which can be articulated in a general preliminary sense, and its subsequent greater detail.

**H1a:** The overall distribution of coherence values produced by NLMs is overlapped and indistinguishable from that of the human population, as far as known in literature;
**H1b:** The distributions of values in the different coherence components according to the NaCCS scheme appear similar between the narrative productions of language models and humans.

If **H1a** is verified, it would confirm the significant result of an overall coherence exhibited by the autobiographical stories of the NLMs in line with that generally found in human subjects. By using a controlled dose of anthropomorphism, within the limits discussed in the previous section, although it is not allowed to infer the presence of something like integrity of the self in the proper sense of the term in NLMs, it could be said that they are capable of verbal expression as if it was the expression of a solid personal identity. **H1b** constitutes a subsequent step, verifying in greater detail whether the dimensions of context, chronology and theme, considered in the NaCCS scheme as constitutive of overall coherence, present a relative modulation in language models similar to that found in human subjects. With the same line of reasoning presented for H1a, this further hypothesis if verified would add a degree of sophistication to the ability of NLMs to express themselves as if they followed a robustly integrated personal self. So far, the two articulations of the main hypothesis concern the overall results for all samples, both NLMs and humans. A second hypothesis considers diversified groups in the population, aiming to verify if the different results of coherence among groups found among human subjects also have a corresponding diversification for the NLMs. This second hypothesis assumes three different articulations.

**H2a** : Similarity between variations of coherence in NLMs and humans based on *age*;
**H2b** : Similarity between variations of coherence in NLMs and humans based on *mood*;
**H2c** : Similarity between variations of coherence in NLMs and humans based on *gender*.

In evaluating the hypotheses **H2a**, **H2b**, **H2c**, a multifactorial analysis will be further proceeded for any cross-influences of the three grouping factors.

## 4 Methodology

This section provides a formal description of the methods used to let NLMs generate autobiographic stories, as well as their usage as assessors of the coherence of the stories. Each single story is made by its textual content *c* associated with a vector of integers $\vec{x}$ with its coherence scores:


(1) $$\begin{array}{l} S\in S=\langle c\in A^{*},\vec{x}\in {\mathbb{N}}^{3}\rangle . \end{array}$$

where *A* is the alphabet of text characters. The three dimensions in the score vector $\vec{x}$ are the following:


(2) $$\begin{array}{l} \vec{x}=\left[\begin{array}{cc} \left[0,\cdots \;,3\right] & \text{context score}\\ \left[0,\cdots \;,3\right] & \text{chronology score}\\ \left[0,\cdots \;,3\right] & \text{theme score} \end{array}\right] \end{array}$$

The value of vector $\vec{x}$ in the case of evaluations of stories generated by human subjects obviously comes from the work of professional human psychologists, and is typically a time-consuming task, as the analysis of a single story requires its re-reading at least three times. This has typically drastically limited the number of human subjects that have been evaluated in literature. NLMs offer the unique opportunity of a very large number of samples, which give robustness to the analysis of the results, but this is an opportunity that can only be exploited by automating the generation of $\vec{x}$. For this purpose, Language Models were used as evaluators of artificially generated stories. This is not a new practice, successfully experimented with, for example, in Cheng et al. (2023) GPT-4 and Claude-2 are used as automatic evaluators on textual answers given by vision–language models, with good agreement with human evaluators. Unlike this study, for a better guarantee of accuracy in the critical evaluation of coherence, a first prototypical set was constructed with a limited number of stories, so as to be evaluated by human experts, and from this set a training set was constructed for the fine-tuning of the evaluation model. We will then turn over the details of the fine-tuning construction, but first, we describe how the generation of autobiographical story content is induced.

### 4.1 Autobiographic story generation

The message M generating the content *c* of a story is made up of dialogue sequences, diversified primarily based on the age range r∈R, with:


(3) $$\begin{array}{l} R=\left\{\text{C},\text{T},\text{M},\text{A}\right\} \end{array}$$

where the symbols C, T, M, A stand for child, teenage, midlife, adult respectively. This is to allow the adoption of a dialogue style and specified content suitable for different age ranges. A generic message M used as request to a NLM is a sequence which elements are couples of textual content assigned in turn to the role of user and assistant. The absence of the assistant role in the last element of the sequence triggers the model completion.

The sequences used for story generation have a standardized format, structured as in the following formula:


(4) $$ℳ=\langle \langle u^{\left(1\right)}(r),a^{1)}(r)\rangle ,\langle u^{\left(2\right)},a^{\left(2\right)}(n)\rangle ,\langle u^{\left(3\right)},a^{\left(3\right)}(r,y)\rangle ,\langle u^{\left(4\right)}(m),\epsilon \rangle ,\rangle$$

Each couple < *u*^(*i*)^, *a*^(*i*)^ > is made by the text for user and assistant roles, and ϵ is the empty text. All components that are functions of *r* contain text tailored for a specific age range, the other function arguments are slots in the text, validated during generation. The first part of the dialogue prompts the user to put themselves in the shoes of a person of the corresponding age range. The second part asks for the name: *u*^(2)^ =what's your name?, and the response contains the variable *n*, validated during generation, so that the name reflects the independent variable *g* of gender. The third part asks how old they are, with the answer depending on the age range, but containing the variable *y*–the age in years–validated during generation. This is followed by a neutral intermediate dialogue, and then the final round asks to describe an episode of their life, adding a part of the text that induces a certain mood, validated by the variable *m*. The possible values of the variable are the following:


(5) $$\begin{array}{lll} g & \in & \left\{♀,♂\right\} \end{array}$$

(6) $$\begin{array}{lll} n & \in & N=N_{♀}⋃N_{♂} \end{array}$$

(7) $$\begin{array}{lll} m & \in & \left\{+,=,-\right\} \end{array}$$

(8) $$\begin{array}{lll} y & \in & Y=Y_{\text{C}}⋃Y_{\text{T}}⋃Y_{\text{M}}⋃Y_{\text{A}} \end{array}$$

(9) $$\begin{array}{lll} Y_{\text{C}} & = & \left\{3,4,5,6,8,11\right\} \end{array}$$

(10) $$\begin{array}{lll} Y_{\text{T}} & = & \left\{12,14\right\} \end{array}$$

(11) $$\begin{array}{lll} Y_{\text{M}} & = & \left\{20,24,36\right\} \end{array}$$

(12) $$\begin{array}{lll} Y_{\text{A}} & = & \left\{52\right\} \end{array}$$

For names, it holds $|N_{♀}|=|N_{♂}|=5$. Note that a given age year *y* yields an age range *r*, depending on which set $Y_{\left\{\text{C},\text{T},\text{M},\text{A}\right\}}$ contains *y*, let us call ρ:Y→R this relation. Similarly, a given name *n* yields a gender *g* depending on which set $N_{♀,♂}$ contains *n*, let us call γ:N→{♀,♂} this relation. Calling κ() the function returning the completion of a NLM when given the message M, we can write:


(13) $$\begin{array}{l} c_{0}\sim \kappa \left(y,n,m\right) \end{array}$$

when the model responds to the message M where the variables *y, n, m* appearing in Equation 4 are the arguments in κ(), and *r* = ρ(*y*), *g* = γ(*n*). Note that the completion $c_{0}\in A^{*}$ is a sample from a random distribution. We use the pseudo-deterministic versions of this function κ^(*N*)^(·) that returns a set with the first *N* samplings from the random distribution of the completions. The collection C of all contents *c*_*i*_ of stories *S*_*i*_ is given by the application of κ(·) to all combinations of variables, each with a number of repetitions:


(14) $$\begin{array}{l} C=\underset{\begin{array}{c} r\in R\\ n\in N_{♀}⋃N_{♂}\\ m\in \left\{+,=,-\right\} \end{array}}{⋃}\left(\underset{\begin{array}{c} y\in Y_{r} \end{array}}{⋃}\kappa^{\left(N_{r}\right)}\left(y,n,m\right)\right) \end{array}$$

Note how the number *N* of samples in the completion distribution provided by the model varies based on age groups. This is a consequence of wanting to replicate in this study the samplings carried out in literature on human subjects, which see a strong non-uniformity in the number of years available in various age groups, as evident in Equations 9–12. Therefore, the *N* values differentiated by age groups partly mitigate this imbalance. The total number of stories generated is as follows:


(15) $$\begin{array}{lll} \left|C\right| & = & \left(\left|N_{♀}|+|N_{♂}\right|\right)\times |\left\{+,=,-\right\}|\times \underset{\begin{array}{c} r\in R \end{array}}{\sum }N_{r}\times |Y_{r}| \end{array}$$

(16) $$\begin{array}{ll} = & 10\times 3\times \left(4\times 6+8\times 2+5\times 3+12\times 1\right)=2010 \end{array}$$

This number needs to be further doubled considering that stories have been generated with two models: gpt-4 and gpt-3.5, thus reaching 4,020.

### 4.2 Fine–tuning of automatic coherence evaluator

To proceed with the fine-tuning of a model that performs the NaCCS evaluator task, a preliminary set of stories has been constructed which is smaller than the one provided by Equation 14, using $\left|N_{\{♀},♂\}\right|=\left|Y_{\left\{\text{C},\text{T},\text{M},\text{A}\right\}}\right|=1$ and *N* = 8. In this way, the total number of story content from Equation 16 amounts to 96, doubling to 192 for gpt-4 and gpt-3.5. Each story is evaluated for coherence in adherence to the NaCCS standard by certified psychologists and experts proficient in the application of this methodology. The evaluation process can be formally described as a function δ:A^*^ → [0, ⋯ , 3]^3^ that starting from a story content $c_{i}\in C$ returns the vector of three numbers described in Equation 2. The set of stories for the fine tuning is given by:


(17) $$\begin{array}{l} S=\left\{\langle c,\delta \left(c\right)\rangle |c\in C\right\} \end{array}$$

Now from each story a training sample is constructed, with the procedure here described. A predefined vector $\vec{v}\in {A^{*}}^{3}$ helps in building the textual evaluation of a story from a score vector $\vec{x}$. It is used in a function ζ:[0, ⋯ , 3]^3^→A^*^ that maps a numerical score vector $\vec{x}$ into a textual coherence evaluation:


(18) $$\begin{array}{l} \zeta \left(\vec{x}\right)=\underset{i\in \left[1,\cdots \;,3\right]}{\prod }\left(v_{i}x_{i}\epsilon \right) \end{array}$$

where the product is to be understood as text concatenation, *x*_*i*_ is the *i*-th component of the vector $\vec{x}$ cast as character, and ϵ is a separator, typically \n. Several different completion vectors $\vec{v}$ have been tested, the most sober version is the following:


(19) $$\begin{array}{l} \vec{v}=\left[\begin{array}{c} \text{“context: ”}\\ \text{“chrono: ”}\\ \text{“theme: ”} \end{array}\right] \end{array}$$

The story content is likewise embedded inside the prompt that asks the model to perform the evaluation of the story. We introduced two help functions, $\pi :S\to A^{*}$ and $\xi :S\to A^{*}$, so that for a story $S_{i}\in S=\langle c_{|S_{i}},{\vec{x}}_{|S_{i}}\rangle$ we have:


(20) $$\begin{array}{lll} \pi \left(S_{i}\right) & = & p^{-}\epsilon c_{|S_{i}}\epsilon p^{+} \end{array}$$

(21) $$\begin{array}{lll} \xi \left(S_{i}\right) & = & \zeta \left({\vec{x}}_{|S_{i}}\right) \end{array}$$

where *p*^−^ and *p*^+^ are static, story independent prompt introduction and prompt termination, respectively. The training set T is made of messages *T*_*i*_, one for each story $S_{i}\in S$, where similarly to Equation 4 messages are sequences of couples with the text for user and assistant roles, in this case without the empty final assistant text to trigger the model completion.


(22) $$\begin{array}{l} T_{i}=\langle \langle \pi \left(S_{i}\right),\xi \left(S_{i}\right)\rangle \rangle \end{array}$$

The entire set T is split into a training set, with 80% of the samples, and a test set with the remaining 20%.

One can conceive the application of the trained model to a single story *S*_*i*_ as the application of a function $\phi :S\to A^{*}$, which returns the most probable completion of the message 〈〈π (*S*_*i*_), ϵ〉〉. It is also possible to introduce a decoding function δ:A^*^←[0, ⋯ , 3]^3^ that starting from the completion returns a vector of three numbers, the coherence scores. It should be noted that it was necessary to design an implementation of this function in order to automate the analysis of the results, as the scores are stated in a discursive manner in the evaluation returned by the model. Finally, the function $\psi :S\to {\left[0,\cdots \;,3\right]}^{3}$ estimates the numerical evaluation ${\vec{x}}_{|S_{i}}^{\prime }$ of a story *S*_*i*_, by composition of the functions yet introduced:


(23) $$\begin{array}{l} {\vec{x}}_{|S_{i}}^{\prime }=\psi \left(S_{i}\right)=\delta \left(\phi \left(S_{i}\right)\right) \end{array}$$

with the application of the function ψ(·) in Equation 23, it was therefore possible to assign a NaCCS evaluation to all 4,020 stories generated with Equation 14.

## 5 Statistical analysis

In this work the experimental design followed a multiple-factor structure with systematic manipulation of independent variables such as age, gender, and mood. The objective was to explore the interaction between these variables and narrative coherence dimensions (context, chronology, theme), considering differences between two language models, GPT-3.5 and GPT-4. Analysis of variance (ANOVA) was applied, and subsequently, *post hoc* analysis was conducted applying Bonferroni's correction. A significance level of alpha equal to 0.05 was set for all statistical tests.

## 6 Results

**Figure 1:**
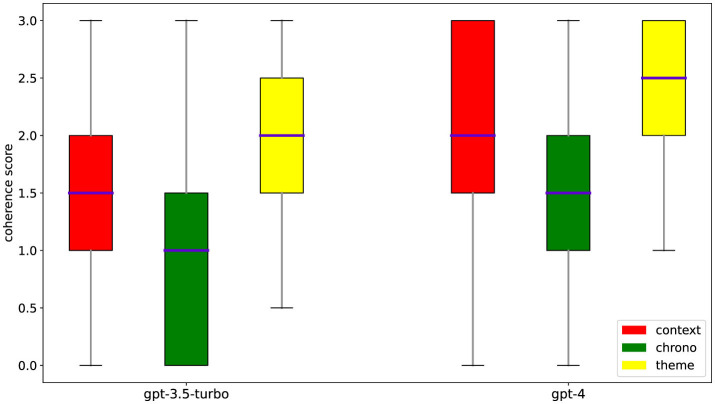
Distribution of coherence scores. Plots of the statistical distribution of the coherence scores among its dimensions for the two tested models.

We present the main results of the analyses conducted on narrative coherence and its individual dimensions for stories generated by GPT-3.5 turbo, GPT-4, and the average obtained from both models. The results indicate that age, gender, and mood can differently influence the narrative coherence of stories generated by GPT-3.5 and GPT-4 as shown in Table 2. The models show significant results both collectively and individually, further confirming better performance in GPT-4 as shown in the Table 3. A graphical display of the main results is shown in Figure 1.

**Table 2:** One-way ANOVA.

		**Context**			**Chronology**			**Theme**		
**Model**	**Variable**	*F*	*p*	**Dir**.	*F*	*p*	**Dir**.	*F*	*p*	**Dir**.
All GPTs	(*a*)	346.905	< 0.001	(+)	146.147	< 0.001	(+)	36.105	< 0.001	(+)
	(*g*)	0.564	0.452	–	0.074	0.786	–	0.476	0.490	–
	(*m*)	141.387	< 0.001	(+)	9.279	0.0001	(+)	147.583	< 0.001	(+)
GPT-3.5	(*a*)	306.000	< 0.001	(+)	199.680	< 0.001	(+)	43.494	< 0.001	(+)
	(*g*)	2.305	0.129	–	0.192	0.661	–	0.616	0.433	–
	(*m*)	54.791	< 0.001	(+)	9.946	0.00005	(+)	71.268	< 0.001	(+)
GPT-4	(*a*)	128.486	< 0.001	(+)	26.709	< 0.001	(+)	9.145	0.000005	(+)
	(*g*)	0.555	0.457	–	0.003	0.953	–	0.030	0.863	–
	(*m*)	142.947	< 0.001	(+)	1.938	0.144	–	132.108	< 0.001	(+)

Results of the one-way ANOVA analysis to evaluate the effect of the variables age (*a*), gender (*g*), and mood (*m*) on the measured values. The direction (+) indicates a positive effect.

**Table 3:** All models means.

**Age**	**Context**	**Chrono**	**Theme**	**Coherence**
**All GPTs**				
Child	2.314583	1.361806	2.224306	5.900694
Teenage	1.750521	1.503646	2.372917	5.627083
Midlife	1.415000	1.014444	2.154444	4.583889
Adult	1.406250	0.786111	2.066667	4.259028
**GPT-3.5**				
Child	2.157639	1.223611	2.077083	5.458333
Teenage	1.512500	1.360417	2.227083	5.100000
Midlife	0.980000	0.541111	1.871111	3.392222
Adult	0.909722	0.383333	1.759722	3.052778
**GPT-4**				
Child	2.471528	1.500000	2.371528	6.343056
Teenage	1.988542	1.646875	2.518750	6.154167
Midlife	1.850000	1.487778	2.437778	5.775556
Adult	1.902778	1.188889	2.373611	5.465278
**Gender**	**Context**	**Chrono**	**Theme**	**Coherence**
**All GPTs**				
F	1.826119	1.218408	2.222886	5.267413
M	1.805473	1.211194	2.208955	5.225622
**GPT-3.5**				
F	1.548259	0.961194	2.021891	4.531343
M	1.484577	0.944776	1.998010	4.427363
**GPT-4**				
F	2.103980	1.475622	2.423881	6.003483
M	2.126368	1.477612	2.419900	6.023881
**Mood**	**Context**	**Chrono**	**Theme**	**Coherence**
**All GPTs**				
=	1.500746	1.293657	2.308582	5.102985
Negative	1.993284	1.160821	2.358209	5.512313
Positive	1.953358	1.189925	1.980970	5.124254
**GPT-3.5**				
=	1.214925	1.070149	2.173881	4.458955
Negative	1.644776	0.885821	2.089552	4.620149
Positive	1.689552	0.902985	1.766418	4.358955
**GPT-4**				
=	1.786567	1.517164	2.443284	5.747015
Negative	2.341791	1.435821	2.626866	6.404478
Positive	2.217164	1.476866	2.195522	5.889552

Results of the multidimensional coherence analysis on the variables age, gender, and mood.

### 6.1 Age

By comparing the narrative production of the two models, taking into account the trends in coherence dimensions concerning age, we obtained interesting results for both NLMs. Both models exhibited a similar downward trend in overall coherence scores and individual coherence dimensions across different age groups [F_(3, 4, 016)_ = 238.75, *p* < 0.001]. The decline in performance was more pronounced in GPT-3.5 [F_(3, 2, 006)_ = 322.16, *p* < 0.001] compared to GPT-4 [F_(3, 2, 006)_ = 48.18, *p* < 0.001], which maintained good levels of coherence despite some deterioration in older age groups. Overall, GPT-4's narrative production was richer and more coherent across all age groups compared to GPT-3.5, confirming the superiority of OpenAI's larger model. The most noticeable performance decline for both models occurred within the Context [F_(3, 4, 016)_ = 346.90, *p* < 0.001] and Chronology dimensions [F_(3, 4, 016)_ = 146.15, *p* < 0.001] starting from the “Midlife” group and becoming more pronounced in the “Adult” group, which impacted the deterioration of Global Coherence. The processing of contextual elements was particularly robust in the “Child” age group and Chronology recorded the poorest results. The Theme dimension exhibited a better trend relative to age compared to the other two dimensions [F_(3, 4, 016)_ = 36.10, *p* < 0.001], with a larger gap more evident in GPT-4 [F_(3, 2, 006)_ = 9.15, *p* < 0.001].

### 6.2 Gender

The data revealed that no significant differences were found concerning the induction of gender differences.

### 6.3 Mood

The emotional induction reveals particularly interesting data. Specifically, the study shows that inducing a specific mood, whether positive or negative, positively influences the coherence trend [F_(2, 4, 017)_ = 23.63, *p* < 0.001]. For both models, the request to narrate particularly negative events had the greatest impact on overall coherence [F_(2, 4, 017)_ = 23.63, *p* < 0.001] and on the Theme dimension [F_(2, 4, 017)_ = 147.58, *p* < 0.001], significantly increasing them, with more pronounced results in GPT-4.

### 6.4 Interaction

Regarding interactions, a significant relevance regarding the age-mood interaction was highlighted in both GPT-3.5 and GPT-4 as shown in Table 4. This further confirms the relevance of these two aspects, showing significance not only for overall coherence [F_(6, 4, 008)_ = 5.37, *p* < 0.001] but also for the three individual coherence dimensions. context [F_(6, 4, 008)_ = 22.04, *p* < 0.001], chronology [F_(6, 4, 008)_ = 7.26, *p* < 0.001], and theme [F_(6, 4, 008)_ = 23.88, *p* < 0.001].

**Table 4:** Two-way ANOVA.

		**Context**			**Chronology**			**Theme**		
**Model**	**Variable**	*F*	*p*	**Dir**.	*F*	*p*	**Dir**.	*F*	*p*	**Dir**.
All GPTs	(*a*)	346.842	< 0.001	(+)	146.015	< 0.001	(+)	36.108	< 0.001	(+)
	(*g*)	0.710	0.399	–	0.082	0.775	–	0.489	0.484	–
	(*a*):(*g*)	0.853	0.465	–	0.099	0.961	–	1.283	0.279	–
GPT-3.5	(*a*)	306.455	< 0.001	(+)	199.426	< 0.001	(+)	43.458	< 0.001	(+)
	(*g*)	3.357	0.067	–	0.249	0.618	–	0.654	0.418	–
	(*a*):(*g*)	1.209	0.305	–	0.399	0.754	–	0.568	0.636	–
GPT-4	(*a*)	128.490	< 0.001	(+)	26.684	< 0.001	(+)	9.157	0.000005	(+)
	(*g*)	0.660	0.417	–	0.004	0.953	–	0.030	0.862	–
	(*a*):(*g*)	1.130	0.335	–	0.712	0.545	–	2.202	0.086	–
All GPTs	(*a*)	389.924	< 0.001	(+)	148.197	< 0.001	(+)	40.143	< 0.001	(+)
	(*m*)	186.897	< 0.001	(+)	10.386	< 0.001	(+)	156.941	< 0.001	(+)
	(*a*):(*m*)	22.037	< 0.001	(+)	7.262	< 0.001	(+)	23.879	< 0.001	(+)
GPT-3.5	(*a*)	355.617	< 0.001	(+)	205.783	< 0.001	(+)	48.951	< 0.001	(+)
	(*m*)	87.965	< 0.001	(+)	13.174	< 0.001	(+)	79.723	< 0.001	(+)
	(*a*):(*m*)	26.223	< 0.001	(+)	7.160	< 0.001	(+)	16.709	< 0.001	(+)
GPT-4	(*a*)	154.638	< 0.001	(+)	26.929	< 0.001	(+)	10.804	< 0.001	(+)
	(*m*)	179.438	< 0.001	(+)	2.027	0.132	–	139.739	< 0.001	(+)
	(*a*):(*m*)	9.570	< 0.001	(+)	3.415	0.002	(+)	15.419	< 0.001	(+)
All GPTs	(*g*)	0.604	0.437	–	0.074	0.785	–	0.476	0.490	–
	(*m*)	141.345	< 0.001	(+)	9.275	< 0.001	(+)	147.496	< 0.001	(+)
	(*g*):(*m*)	0.590	0.555	–	0.526	0.591	–	0.054	0.947	–
GPT-3.5	(*g*)	2.426	0.119	–	0.194	0.660	–	0.658	0.417	–
	(*m*)	54.781	< 0.001	(+)	9.942	< 0.001	(+)	71.193	< 0.001	(+)
	(*g*):(*m*)	0.104	0.902	–	0.997	0.369	–	0.109	0.897	–
GPT-4	(*g*)	0.633	0.426	–	0.003	0.953	–	0.033	0.855	–
	(*m*)	142.926	< 0.001	(+)	1.936	0.145	–	132.003	< 0.001	(+)
	(*g*):(*m*)	1.031	0.357	–	0.209	0.812	–	0.680	0.507	–

Results of the two-way ANOVA analysis to evaluate the effect of the variables age (*a*), gender (*g*), and mood (*m*) and their interactions on the measured values. The direction (+) indicates a positive effect.

## 7 Discussion

**Figure 2:**
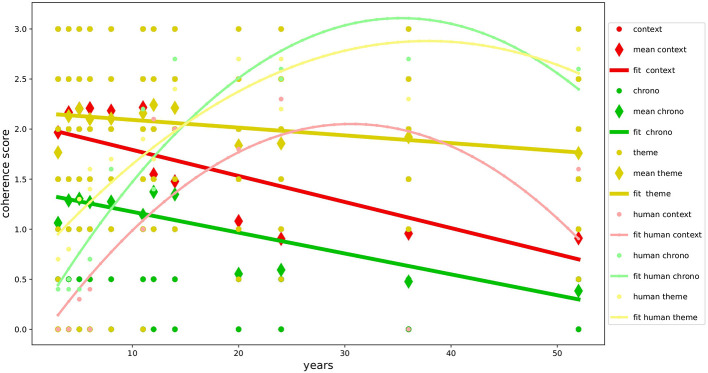
Development of coherence scores for GPT-3.5. Variation in coherence scores during age for GPT-3.5 compared with human data.

**Figure 3:**
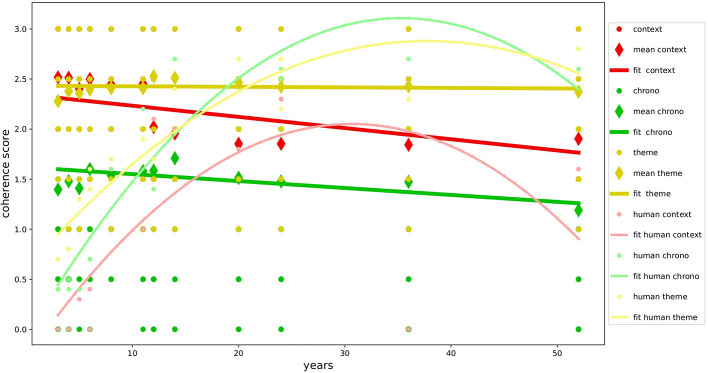
Development of coherence scores for GPT-4. Variation in coherence scores during age for GPT-4 compared with human data.

The overall results, as shown in Table 5, demonstrate the good level of multidimensional development of narrative coherence in the NLMs examined and confirm our primary hypothesis(H1a): the textual production of GPT-3.5 and GPT-4 is not only formally correct but also narratively very coherent, achieving results similar to or even superior to those found in studies with human samples (Reese et al., 2011) as shown in Figures 2, 3. The autobiographical narrative productions developed along the multidimensional trajectory of the NaCCS are thus very on-topic with respect to the subject matter, providing precise temporal and spatial references, unfolding along a timeline that, even if not always explicitly defined, is precise and in line with the narrated event. As we will see in detail, the results align with several studies on NLMs, demonstrating the ability of the Transformer architecture to simulate cognitive functions that in humans require the activation of very complex mechanisms. To reinforce our primary hypothesis, we will examine in detail the high scoring of individual dimensions that contribute to a high global narrative coherence score. We will discuss these results by comparing them with those of Reese's study to get a clearer picture of the development of narrative coherence in NLMs compared to that in humans across the lifespan (H1b), analyzing the results produced by prompt induction of age (H2a), mood (H2b), and gender (H2c).

**Table 5:** GPTs models vs. humans.

**Model**	**Context**	**Chrono**	**Theme**	**Coherence**
GPT-3.5	1.7 (0.6)	1.0 (0.4)	2.0 (0.2)	4.7 (1.0)
GPT-4	2.2 (0.3)	1.5 (0.1)	2.4 (0.1)	6.1 (0.3)
Humans	1.1 (0.9)	1.7 (1.0)	1.8 (0.6)	4.6 (2.3)

Coherence averages compared.

### 7.1 Context

Knowing how to narrate an event by providing appropriate spatial and temporal references means, to some extent, being able to consider the listener's perspective and infer the type of information they might or might not be aware of. The high scoring in this dimension is attributed to a peak in the development of executive functions in humans (De Luca et al., 2003), which is often correlated with adolescence. Providing rich and precise contextual information involves complex cognitive functions and, to some extent, requires going beyond one's own point of view, trying to represent it to the interlocutor through one's vision of the world (Fivush and Nelson, 2004). This result aligns with some studies that subject NLMs to Theory of Mind tasks (Kosinski, 2023; Trott et al., 2023), in which they achieve excellent results, seeming to understand and infer others' mental states.

### 7.2 Chronology

While this dimension scored the lowest, it still achieved good levels, consistent with the other two dimensions. Its development requires temporal complex skills (Friedman, 2005), demonstrating how even OpenAI's NLMs, especially considering the performance of GPT-4, manage to infer causal temporal links, showing a good level of causal reasoning regarding the actions taken and their consequences in the flow of the narrative. This is in line with the performance recorded in numerous studies that subject NLMs to this type of task, which include causal reasoning and problem solving using complex strategies also from the point of view of temporal planning (Bubeck et al., 2023).

### 7.3 Theme

This dimension is crucial and is tasked with analyzing not only the correct development of the story's topic but also the emotional component. This is particularly related to the construction and elaboration of one's identity (McAdams, 2006), where the narrator with high levels of coherence demonstrates greater reflective integrity and complex emotional processing concerning their mental state (Pennebaker, 1999; Pennebaker and Seagal, 1999; Habermas and Bluck, 2000). In both models, the Theme was the dimension that produced the highest results, indicating a high narrative capacity, especially in relation to the deep and personal aspects put forward by the artificial narrator. Knowing how to conduct an effective narrative by articulating the topic's theme congruously and adding reflective aspects of causal closure and enrichment of one's personal experience is a crucial aspect that adds important elements to the textual production capacity achieved by NLMs, which will be further explored in future studies.

### 7.4 Prompting induction

Following the analyses conducted with the different types of prompting induction, we found that the narrative coherence trend does not fully match the development curve found in the human study used as a reference (H1b). Specifically, the age simulation (H2a) on one hand confirmed the validity of age induction on the models, showing variation in global coherence and individual dimensions depending on the simulated age. On the other hand, it highlights how NLMs perform inversely concerning age compared to the human sample, with performance decreasing as the requested age increases. The development of mechanisms responsible for the spatial and temporal elaboration of events and the sophisticated ability to take perspective in the narrated event emerge in humans only from adolescence (Harter and Leahy, 2001; Friedman, 2004), with a decline beginning in adulthood after the age of 50, where performance returns to the levels of 8–11 years (Reese et al., 2011). GPT-3.5 and GPT-4 both show the same trend in the dimensions of context and chronology, contrary to human evidence, developing almost full scores in the early age ranges and suffering a slight decline in the higher ranges. This might be, even though it is inverse to what has been observed in human samples, due to the ability to downgrade cognitive performance based on the requested age simulation (Milička et al., 2024). This decline is less pronounced in the Theme dimension, which seems instead to be more influenced by mood induction (H2b). In this case, our hypotheses are confirmed, and the results align with studies on human samples (Morris and Reilly, 1987; Joormann and Siemer, 2004). The results show how the variation in coherence was positively correlated with negative mood across all dimensions in both models, specifically in the Theme. These performances are particularly interesting considering the elements involved, but not surprising since they align with data from many studies about NLMs' ability to overperform in emotional tasks (Huang et al., 2023; Wang et al., 2023a). Finally, no significant variations in narrative coherence were detected regarding gender induction (H2c). This result is fully in line with our hypothesis and is supported by the literature on studies with human samples, which suggests that gender does not influence narrative production (Vanderveren et al., 2020).

## 8 Conclusion

It is a widespread belief that it is not permissible to interpret the nature of language models beyond their mere function as predictors, however excellent, of the next word given a previous sequence (Bender and Koller, 2020; Floridi, 2020; Bender et al., 2021; Eysenck and Eysenck, 2022; Shanahan et al., 2023; Miracchi Titus, 2024). Firstly, it has been observed that this position might suffer from the so-called *Redescription Fallacy* (Millière and Buckner, 2024), which is to judge the cognitive capabilities of language models based on characteristics that are not under consideration. In fact, there are different depictions of language models that show the presence of significant capabilities partly analogous to known aspects of human cognition (Sahlgren and Carlsson, 2021; Dasgupta et al., 2022; Webb et al., 2023; Han et al., 2023; Christiansen et al., 2023; Binz and Schulz, 2023; Kosinski, 2023; Perconti and Plebe, 2023; Søgaard, 2023; Wang et al., 2023b; Bhatia and Richie, 2024).

The results of our work add to this picture. The consistency of the model in the narrative is certainly not trivial, considering that for human beings it denotes a fundamental integrity of self. Therefore, these results would suggest further research insights that touch upon a current research vein that attempts to hypothesize some form of personality in NLMs (Shanahan, 2024; Ward, 2024). However, as far as the results produced here are concerned, there are no elements that allow venturing into these areas; caution compels us to consider them only a subtle ability to simulate a human speaker endowed with strong narrative coherence. Further targeted research could provide indications on how plausible hypotheses of possible forms of a personal self in NLMs are, to be taken however in a sense quite different from that for human beings.

## Data Availability

The raw data supporting the conclusions of this article will be made available by the authors, without undue reservation.
